# Multilocus Genotypes of Relevance for Drug Metabolizing Enzymes and Therapy with Thiopurines in Patients with Acute Lymphoblastic Leukemia

**DOI:** 10.3389/fgene.2012.00309

**Published:** 2013-01-07

**Authors:** Gabriele Stocco, Raffaella Franca, Federico Verzegnassi, Margherita Londero, Marco Rabusin, Giuliana Decorti

**Affiliations:** ^1^Department of Pharmaceutical Sciences, St. Jude Children’s Research HospitalMemphis, TN, USA; ^2^Department of Life Sciences, University of TriesteTrieste, Italy; ^3^Institute for Maternal and Child Health IRCCS Burlo GarofoloTrieste, Italy; ^4^Scuola di Dottorato di Ricerca in Scienze della Riproduzione, University of TriesteTrieste, Italy; ^5^Ospedale di San Daniele, Azienda per i Servizi Sanitari 4Udine, Italy

**Keywords:** acute lymphoblastic leukemia, mercaptopurine, pharmacogenetics, multilocus genotypes, TPMT, ITPA, PACSIN2

## Abstract

Multilocus genotypes have been shown to be of relevance for using pharmacogenomic principles to individualize drug therapy. As it relates to thiopurine therapy, genetic polymorphisms of *TPMT* are strongly associated with the pharmacokinetics and clinical effects of thiopurines (mercaptopurine and azathioprine), influencing their toxicity and efficacy. We have recently demonstrated that *TPMT* and *ITPA* genotypes constitute a multilocus genotype of pharmacogenetic relevance for children with acute lymphoblastic leukemia (ALL) receiving thiopurine therapy. The use of high-throughput genomic analysis allows identification of additional candidate genetic factors associated with pharmacogenetic phenotypes, such as TPMT enzymatic activity: *PACSIN2* polymorphisms have been identified by a genome-wide analysis, combining evaluation of polymorphisms and gene expression, as a significant determinant of TPMT activity in the HapMap CEU cell lines and the effects of *PACSIN2* on TPMT activity and mercaptopurine induced adverse effects were confirmed in children with ALL. Combination of genetic factors of relevance for thiopurine metabolizing enzyme activity, based on the growing understanding of their association with drug metabolism and efficacy, is particularly promising for patients with pediatric ALL. The knowledge basis and clinical applications for multilocus genotypes of importance for therapy with mercaptopurine in pediatric ALL is discussed in the present review.

## Introduction

The principle of personalized therapy is the identification and application of features associated with treatment response, to select adequate medications and their doses, in order to offer to patients the most effective treatment, with the lower incidence of adverse events (Cheok and Evans, 2006). Among the several features that can be used to personalize therapy, demographic, clinical, and pharmacological ones have been considered. The application of therapy targeted according to these features, related to different treatment outcomes, has greatly improved the effectiveness and safety of therapy, in particular for patients with pediatric cancer, such as acute lymphoblastic leukemia (ALL).

## Personalized Therapy Improves Treatment Effects: The Paradigm of Childhood ALL

Optimal use of existing antileukemic agents and improved supportive care in contemporary clinical trials have improved the 5-year survival rate of childhood ALL above 85% in developed countries, a disease that was universally fatal in the sixties; moreover, molecular characteristics of leukemia cells have been shown to influence treatment response (Pui and Evans, 2006; Pui et al., 2012).

Pharmacological therapy for childhood ALL consists in protocols in which specific treatment approaches may differ but consistently comprise three major treatment phases: remission induction therapy followed by consolidation/intensification therapy and then continuation/maintenance treatment to eliminate residual leukemic cells (Pui and Evans, 2006). Several medications are used in these treatment phases, comprising various lympholytic and cytotoxic drugs such glucocorticoids (i.e., prednisone, dexamethasone), asparaginase and vincristine, which are particular important for the induction of disease remission. The purine analog mercaptopurine is a key medication for the successful treatment of childhood ALL, in particular for the consolidation and continuation therapies and is used in combination with the folate analog methotrexate: for the success of ALL treatment, the 18–24 months of adequate maintenance therapy comprising mercaptopurine and methotrexate have a key role and are necessary to prolong and consolidate the remission obtained during the initial treatment phases (Pui and Evans, 2006; Paugh et al., 2010; Stocco et al., 2010).

### Genetic features may influence response to therapy

Genetic polymorphisms for genes involved in drug metabolism, transport and molecular mechanism of action can alter the concentration of active metabolites and the molecular function of drugs’ targets and therefore the efficacy and safety of pharmacological therapies (Paugh et al., 2011; Pinto et al., 2012). These genetic polymorphisms could therefore function as biomarkers for toxicity and efficacy, allowing the identification of patients with modified sensitivity, because of their genetic characteristics involving drug pharmacokinetics and pharmacodynamics. While many associations between single genetic polymorphisms and drug effects have been clearly demonstrated, showing that inherited genomic variation causes substantial interindividual differences in drug effects, the clinical implementation of these associations is still limited (Relling and Klein, 2011). This is due mainly to the lack of freely available, peer-reviewed, updatable, and detailed gene/drug clinical practice guidelines and even to the very high standards many clinicians and regulators hold for pharmacogenetic evidence (Relling et al., 2010). One of the main efforts to provide these guidelines, which could base the clinical implementation of pharmacogenomics, is that of the Clinical Pharmacogenetics Implementation Consortium (CPIC; Relling and Klein, 2011). CPIC was established in 2009 and consists of members of the Pharmacogenomics Research Network, the main US-based research network in this field, supported by the PharmGKB staff, one of the most important resources for curated pharmacogenomics knowledge (McDonagh et al., 2011), and other affiliated experts in pharmacogenetics, pharmacogenomics and laboratory medicine (Relling and Klein, 2011).

Currently, the CPIC has provided guidelines for pharmacogenetic implementation for 7 medications: abacavir, allopurinol, clopidogrel, codeine, simvastatin, thiopurines, and warfarin (https://www.pharmgkb.org/page/cpic). The process of guidelines definition and preparation is still ongoing and other potential guidelines may be of interest, such as inosine triphosphate pyrophosphatase (ITPA) genetic polymorphism and ribavirin (Fellay et al., 2010), for which a good amount of evidence and replication has been made (Ochi et al., 2010; Thompson et al., 2010; D’Avolio et al., 2012). Moreover, besides CPIC, other research groups have been putting together similar guidelines, such as the European Dutch and German translational pharmacogenomics research teams (Swen et al., 2008, 2011; Schwab and Brauch, 2012).

For the pharmacological therapy of pediatric ALL, several examples have been reported of genetic polymorphisms influencing drug response and toxicity, such as for prednisone polymorphisms of *SMARCB1* (Pottier et al., 2007, 2008) and *GST-M1* (Marino et al., 2009), for methotrexate solute carrier organic anion transporter family member 1B1 (SLCO1B1) (Trevino et al., 2009; Ramsey et al., 2012), for vincristine *ABCB1* and *CYP3A4*/*CYP3A5* (Paugh et al., 2010); however, the only drug – gene pair with a validated guideline published by CPIC that is fully relevant for therapy of pediatric ALL is that of mercaptopurine and thiopurine-*S*-methyltransferase (TPMT). Indeed, for mercaptopurine, genetic polymorphisms of *TPMT* have been demonstrated to influence drug metabolism and its effects, constituting one of the most studied and significant example of associations between drug clinical effects and a genetic polymorphism (Paugh et al., 2011). In lymphoid tissues, mercaptopurine is converted to its active metabolites, the thioguanine nucleotides (TGNs) and is inactivated primarily to methylmercaptopurine by TPMT (Stocco et al., 2010; Zaza et al., 2010). TPMT is encoded by a gene that has non-synonymous single-nucleotide polymorphisms, leading to reduced TPMT activity. In the majority of world populations studied to date, ∼1 in 180 to 1 in 3,700 individuals (depending on ethnicity) inherit two non-functional variants of the *TPMT* gene, 3–14% are heterozygous, and the rest are homozygous wild-type. With chronic conventional doses of mercaptopurine, patients who inherit two inactive *TPMT* alleles universally experience severe myelosuppression, because of accumulation of high levels of cellular TGNs; a high proportion (30–60%) of patients heterozygous for a *TPMT* variant allele does not tolerate full doses of mercaptopurine, again because of excessive TGNs. Three *TPMT* single-nucleotide polymorphisms account for more than 90% of inactivating alleles and therefore genotyping tests have a high likelihood of being informative. Characterization of TPMT deficiency by genotyping for the most common inactivating single-nucleotide polymorphisms can prospectively identify patients at higher risk of mercaptopurine hematopoietic toxicity; such genotyping is recommended in US Food and Drug Administration-approved labeling (Paugh et al., 2010, 2011).

The diagnosis of TPMT deficiency allows the rational reduction of mercaptopurine dosages while other concurrent cytotoxic agents remain at their usual unadjusted doses, thereby avoiding toxicity without compromising efficacy. For patients with ALL taking mercaptopurine, the CPIC guidelines indicate that these subjects with the homozygous variant should start with drastically reduced dose (i.e., reduce daily dose 10-fold and reduce frequency to thrice weekly instead of daily) and in case of myelosuppression, emphasis should be on reducing mercaptopurine over other agents; patients heterozygous for *TPMT* variant alleles (intermediate activity) should start at 30–70% of full dose and again, in case of myelosuppression, emphasis should be on reducing mercaptopurine over other agents. Patients with normal *TPMT* should begin therapy with normal starting dose and adjust doses of mercaptopurine (and of any other myelosuppressive therapy) without any special emphasis on mercaptopurine compared to other agents. For all genotypes, in case of dose adjustment, the guidelines specify to allow 2–6 weeks to reach steady state after each dose adjustment, with longer time needed for patients with inactive allele(s) as compared to patients with functional ones (Relling et al., 2011; Table 1). Indeed, it has been shown that, in an ALL protocol using mercaptopurine, prospective adjustment of mercaptopurine based on TPMT status allowed successful treatment of patients with variant *TPMT* at a reduced dose, with toxicity and efficacy comparable to those in patients with wild-type *TPMT* (Relling et al., 2006; Stocco et al., 2009).

**Table 1:** **Recommended dosing of thiopurines by thiopurine methyltransferase phenotype**.

TPMT status	Mercaptopurine		Thioguanine	
	Effects on mercaptopurine metabolism	Dosing recommendations for mercaptopurine	Effects on thioguanine metabolism	Dosing recommendations for thioguanine
Homozygous wild-type or normal, high activity	Lower concentrations of TGNs metabolites, higher methylTIMP, this is the “normal” pattern	Start with normal starting dose (e.g., 75 mg/m^2^/day) and adjust doses of mercaptopurine (and of any other myelosuppressive therapy) without any special emphasis on mercaptopurine compared to other agents	Lower concentrations of TGNs metabolites, but note that TGNs after thioguanine are 5–10× higher than TGNs after mercaptopurine	Start with normal starting dose. Adjust doses of thioguanine and of other myelosuppressive therapy without any special emphasis on thioguanine.
Heterozygote or intermediate activity	Moderate to high concentrations of TGNs metabolites; low concentrations of methylTIMP	Start with reduced doses (start at 30–70% of full dose: e.g., at 50 mg/m^2^/day or 0.75 mg/kg/day) and adjust doses of mercaptopurine based on degree of myelosuppression and disease-specific guidelines. In those who require a dosage reduction based on myelosuppression, the median dose may be ∼40% lower (44 mg/m^2^/day) than that tolerated in wild-type patients (75 mg/m^2^/day). In setting of myelosuppression, and depending on other therapy, emphasis should be on reducing mercaptopurine over other agents	Moderate to high concentrations of TGNs metabolites; but note that TGNs after thioguanine are 5–10× higher than TGNs after mercaptopurine	Start with reduced doses (reduce by 30–50%) and adjust doses of thioguanine based on degree of myelosuppression and disease-specific guidelines. In setting of myelosuppression, and depending on other therapy, emphasis should be on reducing thioguanine over other agents
Homozygous variant, mutant, low, or deficient activity	Extremely high concentrations of TGNs metabolites; fatal toxicity possible without dose decrease; no methylTIMP metabolites	Start with drastically reduced doses (reduce daily dose by 10-fold and reduce frequency to thrice weekly instead of daily, e.g., 10 mg/m^2^/day given just 3 days/week) and adjust doses of mercaptopurine based on degree of myelosuppression and disease-specific guidelines. In setting of myelosuppression, emphasis should be on reducing mercaptopurine over other agents	Extremely high concentrations of TGNs metabolites; fatal toxicity possible without dose decrease	Start with drastically reduced doses (reduce daily dose by 10-fold and dose thrice weekly instead of daily) and adjust doses of thioguanine based on degree of myelosuppression and disease-specific guidelines. In setting of myelosuppression, emphasis should be on reducing thioguanine over other agents

*TGNs, thioguanine nucleotide; TIMP, thioinosine monophosphate (secondary metabolite of mercaptopurine); TPMT, thiopurine-*S*-methyl transferase (Relling et al., 2011)*.

### Multilocus genotypes

Most of the associations evaluated in the literature and those ready for clinical implementation, i.e. with published curated guidelines, are considering single gene influencing response to a particular drug. However, one of the most important, in terms of relevance and diffusion of drug treatment and potential improvement of guideline application to influence drug use in the clinical setting are the guidelines for warfarin, the most commonly used oral anticoagulant worldwide (Johnson et al., 2011); indeed, these guidelines consider genetic variability at two loci: one for the hepatic drug metabolizing enzyme CYP2C9 and one for the target enzyme of warfarin, that is vitamin K-epoxide reductase (VKORC1). *CYP2C9* and *VKORC1* genetic polymorphisms account for up to 18 and 30%, respectively, of the variance in stable warfarin dose among patients of European ancestry: these common polymorphisms in both genes affect warfarin pharmacokinetics (*CYP2C9*) and pharmacodynamics (*VKORC1*) and modulate the therapeutics dose necessary to maintain the optimal level of drug effect (i.e., anticoagulation), preventing the risk of adverse events due to low efficacy or excessive anticoagulation (i.e., respectively thrombosis or bleeding). Combination of *CYP2C9* and *VKORC1* genetic polymorphisms is important to select the most appropriate dose to start therapy with warfarin: patients are classified by the multilocus genotype in a 2 × 2 table, according to the combined effects of the most relevant polymorphisms in each gene (two SNPs for *CYP2C9* and one SNP for *VKORC1*) in three levels of warfarin starting dose; this table is currently inserted in the US Food and Drug’s Administration approved warfarin label. More complex algorithms, comprising even relevant demographic and clinical patient’s characteristics affecting warfarin efficacy, such as age, smoking status and interacting drugs have been developed; some of these algorithms consider even additional genetic information besides *CYP2C9/VKORC1* multilocus genotype, such as polymorphisms of the *CYP4F2* and *GGCX* genes. It has been shown that warfarin dosing criteria considering genetics outperform non-genetic clinical algorithms and are particularly beneficial for patients requiring relatively low or high doses of the medication (i.e., <21 mg/week or >49 mg/week), that however are ∼40% of all patients: thanks to genetic based dose selection, these patients reach their optimal dose level more quickly and therefore with a lower risk of developing adverse events. Moreover, these criteria are particularly important for patients starting warfarin therapy, while are less useful for already established treatments. The development of these important therapeutic guidelines, considering a multilocus genotype affecting warfarin dose requirements illustrates how genetic information in more than one gene can be of clinical relevance to guide therapy for a single medication. Other similar guidelines considering multiple loci are in development, such as *CYP2D6*/*CYP2C9* multilocus genotype for tricylic antidepressants (Consortium, 2012).

## Multilocus Genotypes of Relevance for Therapy Personalization of Pediatric ALL

### TPMT and ITPA and maintenance therapy for pediatric ALL

In addition to *TPMT*, other genetic factors may alter the effects of mercaptopurine, although their clinical importance has not been as well characterized. It has been shown that once mercaptopurine treatment for childhood ALL is individualized for *TPMT*, the effect of genetic polymorphisms in inosine triphosphate pyrophosphatase (ITPA) emerges (Stocco et al., 2010). ITPA is an enzyme that catalyzes the hydrolysis of inosine triphosphate (ITP) to inosine monophosphate (IMP). IMP is a central intermediate in purine metabolism and can be converted to ITP and to ATP via AMP or to GTP via GMP. The putative role of ITPA is to protect cells from the accumulation of potentially harmful nucleotides, such as ITP or deoxy-ITP, which may be incorporated into nucleic acids; indeed, it has been demonstrated by knock-down experiments performed in HeLa cells, that ITPA has a significant role in preventing base analog induced apoptosis, DNA damage, and mutagenesis in human cells (Menezes et al., 2012). In humans, ITPA displays a genetically determined polymorphic activity (Marsh and Van Booven, 2009; Stocco et al., 2010). Characterization of *ITPA* haplotype structure has shown that the SNP rs1127354 is the most relevant polymorphism in determining ITPA low enzymatic activity (von Ahsen et al., 2008; Stocco et al., 2010). Our recent study assessed the influence of non-functional variant alleles of *TPMT* and *ITPA* on mercaptopurine metabolism and toxicity in patients with ALL whose mercaptopurine doses were adjusted based on *TPMT* genotype (Stocco et al., 2009). This study revealed that the cumulative incidence of severe adverse effects (grade 3–4 febrile neutropenia) in patients receiving maintenance therapy that includes mercaptopurine individualized for TPMT is significantly greater among patients who have inherited an *ITPA* variant allele; this association remained significant when the analysis was limited to only life threatening events (i.e., grade 4 fever and neutropenia). Our recent study has documented that inheritance of a non-functional variant allele for either *TPMT* or *ITPA* is associated with significant modification in the metabolism of mercaptopurine during treatment of ALL. Although the importance of the *TPMT* genetic polymorphism is very well known and characterized, this was the first report showing a significant effect of the *ITPA* genetic polymorphism in the context of mercaptopurine therapy that has been individualized based on *TPMT* genotype. We documented significantly higher concentrations of the methylated nucleotide metabolites of mercaptopurine in leukemia cells and erythrocytes of patients who have inherited a non-functional *ITPA* allele. In contrast, the inheritance of a variant *ITPA* allele was not associated with differences in TGN concentrations in either leukemia cells or erythrocytes. Although ITPA is known to be involved in mercaptopurine metabolism, the mechanism by which ITPA variant alleles influence the accumulation of methylated thionucleotides has not been fully elucidated (Stocco et al., 2009, 2010).

A recent study has replicated the observation of the effects of the combined *TPMT* and *ITPA* genotype on the mercaptopurine pharmacokinetics and in particular on the concentration of methylated-mercaptopurine-nucleotides: among 66 children with ALL, treated according to EORTC 58951 protocol, comprising mercaptopurine at a dose of 50 mg/m^2^/day and methotrexate at a dose of 20 mg/m^2^/week, methylated-mercaptopurine-nucleotides concentrations were low in patients with *TPMT* variant/*ITPA* wild-type multilocus genotype, intermediate in wild-type/wild-type patients and high in patients with wild-type *TPMT*/*ITPA* variant (Adam de Beaumais et al., 2010).

It is known that ethnic differences for genotype frequencies may influence treatment efficacy in ALL: for example, it has been reported that the component of genomic variation that co-segregated with Native-American ancestry was associated with risk of relapse, even after adjusting for known prognostic factors (Yang et al., 2011). The allele frequencies of *TPMT* and *ITPA* polymorphisms show significant inter-ethnic variability: in particular for rs1127354 of *ITPA* allele frequency of the variant is known to be ∼20% in Asian populations, ∼6% in Caucasians, and ∼2% in Hispanics, while for *TPMT*, the most common variants (rs1142345, rs1800460 and rs1800462) have a frequency of ∼1% in Asians, ∼5% in Caucasians, and ∼10% in Hispanics. Therefore, it is interesting that for *TPMT* and *ITPA*, frequencies of the variant alleles associated with different metabolism of mercaptopurine, seem to be almost reversal in the two populations (Marsh and Van Booven, 2009) and *ITPA* variants seem to be predominant in the Asian population. Indeed several recent studies of patients of Asian ethnicity seem to underline significant effects of *ITPA* polymorphisms on thiopurines’ efficacy and toxicity in patients with ALL, but even when these medications are used as immunosuppressants in other pathologies (Okada et al., 2009; Yamamoto et al., 2010). For children with ALL, a recent study in 90 Indian patients, on maintenance therapy according to the MCP-841 protocol (Advani et al., 1999) with mercaptopurine at a dose of 75 mg/m^2^ for 12 weeks, showed an independent role for both *TPMT* and *ITPA* in terms of association with the incidence of hematological toxicity; moreover, the multilocus genotype *TPMT*/*ITPA* was associated with a gene-dosage effect: percentage of reduction in total leukocyte count (i.e., the average leukocyte count on days 43, 71, and 99 of maintenance therapy) resulted in ∼40% for a patient with a wild-type genotype at both the *TPMT* and *ITPA* loci and increased proportionally to the number of risk alleles (i.e., variant inactive alleles for *TPMT* or *ITPA*) up to almost 70% in patients with three or more risk alleles at the *TPMT* and *ITPA* loci (Dorababu et al., 2012a). Analysis of epistasis by multifactor dimensionality reduction (Hahn et al., 2003) confirmed synergistic interactions between *TPMT* and *ITPA* variant alleles, in terms of their association with hematological toxicity during ALL maintenance therapy for this cohort of Indian children. Another recent study considered 100 Korean patients with pediatric ALL and evaluated in these patients 18 loci in 16 candidate genes of pharmacogenetic interest, including *TPMT* and *ITPA*, and their association with survival rate. Even if this study did not seem to confirm a strong difference for TPMT and ITPA gene variants between a western population of reference and the Korean patients, there was a significant effect of *ITPA* genotype, but not of *TPMT*, on the event free survival rate, which was lower in *ITPA* variants. *TPMT* genotype was however associated with the tolerance of mercaptopurine and methotrexate, evaluated as the dose of the medications used during the last cycle of maintenance therapy: indeed, as expected, patients with variant *TPMT* were selected to be treated with lower doses of mercaptopurine; unfortunately, data about the effect of *ITPA* genotype on the doses of antimetabolites was not reported (Kim et al., 2012).

Tanaka et al. (2012) have measured the activity of ITPA in 65 Japanese children with pediatric ALL, showing that patients with lower activity of this enzyme tolerated lower doses of mercaptopurine during maintenance therapy and presented increased probability of hepatoxicity.

In Asian populations, therefore, polymorphisms of *ITPA* seem to be of particular relevance for the effects of mercaptopurine in children with ALL, given the low incidence of patients with variant *TPMT*, compared to patients of Caucasian ethnicity (Marsh and Van Booven, 2009). However, it is known that other genetic polymorphisms may be of particular importance for Asian patients, such as SNP rs3765534 in the transporter MRP4, that is polymorphic only in patients of Asian ethnicity and that has been shown to modulate thiopurines intracellular levels by regulating the efflux of the thionucleotides (Krishnamurthy et al., 2008; Stocco et al., 2010).

On these bases, to understand the pharmacogenetics and improve treatment with thiopurines in the Asian populations, larger prospective studies are needed, considering even multilocus genotypes at loci of known relevance, such as *TPMT*, *ITPA*, and *MRP4*.

### Multilocus genotype *TPMT* – *SLCO1B1* – *PACSIN2* and effects on severe mucositis during consolidation therapy for pediatric ALL

During consolidation therapy for pediatric ALL, patients are treated with weekly 24 h infusions of high dose methotrexate, up to 5 g/m^2^ and daily oral mercaptopurine with doses that range from 25 to 50 mg/m^2^. Therapy with this association of antimetabolites has a very important role in preventing the relapse of the disease, after remission induction; however consolidation therapy is associated with the development of adverse effects, in particular gastrointestinal toxicity, such as stomatitis and mucositis, which cause major discomfort for the patient and can be severe, preventing the children from normal food intake and requiring parenteral nutrition. To avoid adverse events related to consolidation therapy, one of the most common approaches used in therapeutic protocols for ALL worldwide is the administration of leucovorin, a source of folic acid, that contrasts the cytotoxic effects of methotrexate and its association with mercaptopurine. Most protocols for ALL worldwide measure the concentration of methotrexate in patients’ blood at the end of each infusion and administer leucovorin if methotrexate is then still present at significant concentrations: for example, in the Italian AIEOP-BFM ALL 2000 protocol, leucovorin was administered every 6 h at a dose of 7.5 mg/m^2^, if methotrexate concentration resulted higher than 0.5 μmol/l at 48 h from the beginning of the infusion and until methotrexate concentration dropped below 0.25 μmol/l (Conter et al., 2010; Schrappe et al., 2011). Consolidation therapy lasts from 2 weeks up to 3 months depending on the treatment protocol and therefore the length of the therapy is too short to implement therapeutic monitoring of mercaptopurine metabolites concentration, which are useful when the drug is taken for at least 2 months (Lennard and Lilleyman, 1989). Advanced protocols for treatment of ALL, developed at St. Jude Children’s Research Hospital in Memphis, evaluate the clearance of methotrexate during the infusion and either adjust the speed of infusion of the drug to a target concentration in the subsequent course (Total XV protocol) or in the same course (Total XVI protocol). This procedure requires a quick and efficient turnaround of the samples for the measurement of methotrexate concentration, which need to be analyzed in a few hours timeframe, so that the clearance of methotrexate can be estimated during the infusion, and the medication’s administration speed can be adapted to reach the desired concentration threshold (i.e., 33 μM for low risk patients, 65 μM for standard-high risk patients; Pui et al., 2009). Therapeutic monitoring of methotrexate during consolidation therapy has significantly improved patients’ tolerance to this association treatment with antimetabolites; however, about 5% of pediatric patients still develop severe stomatitis/mucositis, with consequences that can be life threatening. The study of pharmacogenetic determinants of severe gastrointestinal (GI) toxicity during consolidation therapy has lead to major breakthroughs in recent years, which hopefully will lead to even better treatment of patients with ALL, completely preventing the occurrence of this major adverse event. In particular, a recent genome-wide study analyzed 500,568 germline single-nucleotide polymorphisms to identify how inheritance affects methotrexate plasma disposition among 434 children with ALL who received 3,014 courses of methotrexate at 2–5 g/m^2^ (Trevino et al., 2009). This study lead to the identification of polymorphisms in *SLCO1B1*, as the most significant associations (*p*-value < 10^−9^) with methotrexate clearance, even after adjusting for age, race, sex, and methotrexate regimen. In particular, the most significant polymorphism was the intronic rs11045879, which is in linkage disequilibrium with the functional SNP rs4149056; these same polymorphisms were associated even with severe GI toxicity during consolidation therapy, mostly severe stomatitis and mucositis. This observation was confirmed by subsequent studies (Lopez-Lopez et al., 2011; Ramsey et al., 2012). Therefore *SLCO1B1* polymorphisms are significant determinants for the occurrence of severe GI toxicity and in particular stomatitis/mucositis, during consolidation therapy for pediatric ALL, by an effect on methotrexate disposition: indeed the gene product of *SLCO1B1* is a transporter which mediates the sodium-independent uptake of organic anions such as methotrexate and may play an important role in the clearance of bile acids and organic anions.

**Figure 1:**
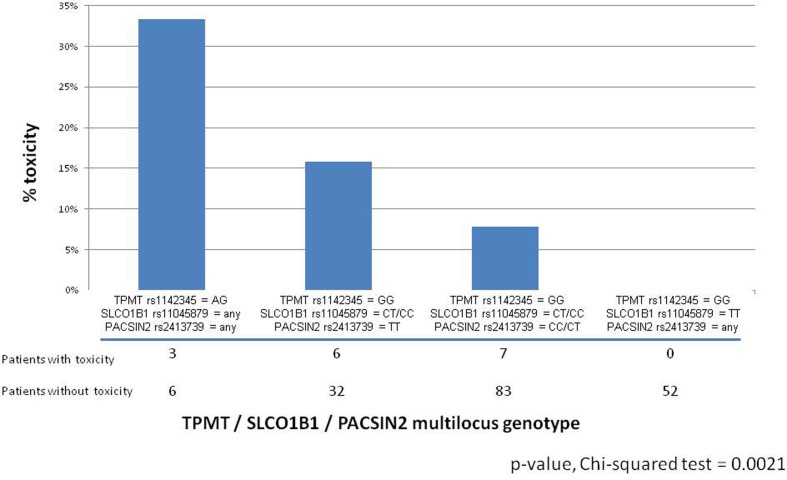
**Barplot reporting the percentage of patients developing severe (Grade 3–4) GI toxicity during consolidation therapy in patients with ALL treated according to the St Jude Total 13B protocol as a function of TPMT rs1142345 / SLCO1B1 rs11045879 / PACSIN2 rs2413739 multilocus genotype (Stocco et al., 2012)**.

Another relevant study has considered the role of genetic determinants of mercaptopurine toxicity during consolidation therapy, together with *SLCO1B1*: this study on adverse effects considered 189 children with ALL and evaluated the association between genetic determinants of *TPMT* activity in patients and the incidence of severe GI toxicity. The frequency of GI toxicity (grade 3–4 mucositis) in this population was 8.5%; among these patients, deficiency in TPMT activity predisposed to an increased incidence of severe GI toxicity during consolidation therapy which included methotrexate (2 g/m^2^/week) and mercaptopurine (75 mg/m^2^/day, in all patients during consolidation therapy, regardless of TPMT genotype). Indeed, among nine patients with a variant TPMT allele, the frequency of GI toxicity was 33%, compared with 7.2% in patients with wild-type TPMT. As previously reported (Trevino et al., 2009), the *SLCO1B1* SNP rs11045879 was also associated with the incidence of GI toxicity: indeed none of the patients with the *SLCO1B1* CC or CT genotype had GI toxicity, whereas 11.8% of the patients with the wild-type *SLCO1B1* TT genotype had this side effect. Moreover, this study identified, through the HapMap model system, an additional determinant of TPMT activity, the *PACSIN2* gene which resulted as the highest correlated gene to TPMT activity, in an analysis combining polymorphisms and expression, all measured in the 30 HapMap CEU trios (Stocco et al., 2012). The most significant *PACSIN2* SNP in the HapMap analysis for TPMT activity, rs2413739, was also significantly associated with TPMT activity in patients with ALL, independently from *TPMT* genotype: the CC genotype for the rs2413739 SNP displayed a higher TPMT activity in comparison to the TT genotype. Moreover, *PACSIN2* SNP rs2413739 also had a significant association with GI toxicity during consolidation therapy: the frequency of toxicity was 2.1, 9.1, and 13.2%, respectively, for the CC, CT, and TT genotype (Stocco et al., 2012). The effects of *PACSIN2* polymorphism on the incidence of severe mucositis during consolidation therapy for pediatric ALL were confirmed in another cohort of patients, considering 67 cases developing the adverse event during therapy according to the protocol AIEOP-BFM ALL 2000, which involves four weekly infusion of methotrexate at the dose of 2 g/m^2^ and concomitant daily treatment with mercaptopurine at the dose of 25 mg/kg. Analysis in the validation cohort was done by a case-control design and each case was matched to two controls from the same protocol based on sex, age, ALL lineage and ALL risk classification, confirming a significant effect of *PACSIN2* SNP rs2413739 on the incidence of severe mucositis during the consolidation therapy of pediatric ALL (Stocco et al., 2012). Interestingly, in the discovery cohort, the effects of *TPMT*, *SLCO1B1* and *PACSIN2* polymorphisms were independent from each other, both in a multivariate logistic regression model and in a classification and regression tree analysis and could be combined in a multilocus genotype of potential importance to predict the incidence of severe mucositis in children with ALL treated with consolidation therapy comprising the combination of methotrexate and mercaptopurine (Figure 1).

### Genome-wide analysis of SNPs associated to clinical response in pediatric ALL: Implications for the pharmacogenetics of mercaptopurine

Genome-wide analysis, if adequately powered, has great potential in elucidating and understanding the genomic component associated with interindividual differences in phenotypes, even of pharmacogenetic interest. This has been shown to be true in model systems like the HapMap cell lines, in which statistical power is obtained mainly by combining genomic information at the level of gene expression and genetic polymorphisms, with the advantage that the phenotypes can be characterized with great accuracy and consistency (Wheeler and Dolan, 2012). This has led for example to the identification of *PACSIN2* as a significant determinant of TPMT activity in the cell lines, with effects reproducible in patients with ALL mentioned above (Stocco et al., 2012). However, the greatest potential of the genome-wide approach resides really in the analysis of patients’ samples: if the study is adequately designed and powered and the phenotypes are well collected, this approach can provide unpredictable insights on the phenotype of interest, potentially leading to major breakthroughs in the understanding of the genomic basis of inter-patient variability, even of pharmacogenetic traits. Several such examples exist in the literature: besides the already mentioned role of SLCO1B1 in the disposition of methotrexate in children with ALL (Trevino et al., 2009), this same transporter was shown to be involved in statins’ induced myopathy (Link et al., 2008); another example of genome-wide studies is the discovery of a role for ITPA in anemia induced by the anti-viral agent ribavirin (Fellay et al., 2010).

While in pediatric ALL genome-wide interrogation is complicated by the relative small number of patients available, St. Jude Children’s Hospital was able to publish some genome-wide studies on leukemia pharmacogenetics, thanks to access to uniformly treated and well characterized patients and phenotypes (Trevino et al., 2009; Yang et al., 2009, 2012; Kawedia et al., 2011). Among these genome-wide studies of pharmacogenetic interest, some have generated data that could be of particular interest for the identification of multilocus genotypes of relevance for the treatment of ALL with thiopurines. In particular, these studies considered genetic polymorphisms associated with outcome to therapy evaluated as minimal residual disease (MRD) (Yang et al., 2009) or disease relapse (Yang et al., 2012); while these very important clinical phenotypes of patients with ALL are not related directly only to mercaptopurine, the genetic features identified are related even to disposition of antileukemic drugs and may be of relevance for mercaptopurine effects too and should be therefore considered.

The study on MRD considered two independent cohorts of children with newly diagnosed ALL: 318 patients in St Jude Total Therapy protocols XIIIB and XV and 169 patients in Children’s Oncology Group trial P9906. This study identified 102 SNPs associated with MRD in both cohorts, including five SNP in interleukin 15 (*IL15*). Twenty one of these SNPs were also associated with drug disposition (evaluated as methotrexate clearance, etoposide clearance, or methotrexate polyglutamates concentration), generally linking greater drug exposure with MRD eradication. While concentration of mercaptopurine metabolites was not evaluated in this study, the effects on the disposition of methotrexate, that is associated with mercaptopurine both during consolidation and maintenance therapy, suggest that these SNPs may be of interest to build multilocus genotypes useful for therapy personalization of pediatric ALL also with mercaptopurine.

### Further development of multilocus genotypes: Epistasis and gene-environment interactions

Phenotypes of pharmacogenetic interest are complex, particularly those describing patients’ response to a medication, both in terms of efficacy and incidence of adverse events: it is likely that different genetic features, together with environmental factors, contribute to the interindividual variability of these phenotypes. Indeed, it is known that the effect of genetic polymorphisms is stronger when it refers to a pharmacokinetic phenotype and strength of the association reduces with the increasing complexity of the phenotype: for example, the effect of *TPMT* genotype is extremely strong on TPMT activity and the strength of the association is reduced, while still significant, considering more complex phenotypes such as the concentration of mercaptopurine metabolites and, even more, considering parameters of clinical response to the medication: the thinning of the association strength is due to the increasing complexity of the phenotype and the augmented potential role of environmental and additional genetic factors (Relling et al., 2011). Moreover, for complex phenotypes such as the response to a medication, the effects of a genetic factor may depend on other genetic variations and environmental factors, a phenomenon that is defined respectively as gene-gene interaction/epistastis or as gene-environment interaction (Moore and Williams, 2009). Methods have been developed to study consistently and efficiently the role of multiple genetic and environmental factors on complex phenotypes defined by discrete traits, such as those of pharmacogenetic interest (e.g., clinical response to a medication or occurrence of adverse events; Hahn et al., 2003; Gilbert-Diamond and Moore, 2011). This method is called multifactor dimensionality reduction (MDR) and allows collapsing multi-dimensional genetic information into a single dimension, thus permitting the detection of epistasis: MDR is a non-parametric method and interactions are detected by a constructive induction approach, in particular by classifying multiple loci as high risk or low risk, depending on whether they are more common in affected or in unaffected subjects; this pooling allows reducing the dimensionality of the multilocus data to one dimension (Hahn et al., 2003). The new multilocus genotype variable is then evaluated for its ability to classify and predict the phenotype of interest (i.e., drug response): different approaches have been used to perform these computations; originally, however, it was done by cross-validation and permutation testing and recently extensions and variations of the method have been developed, which allow the calculation of odds ratios and application of Fisher’s test to increase model robustness (Moore and Williams, 2009). Interestingly, it has been reported that MDR allows the identification of significant gene–gene interaction in the absence of a statistically significant main effect by a single genotype; moreover, it was mathematically proved that MDR is the best method to discriminate multilocus genotypes for clinical endpoints. MDR has been successfully applied to detecting gene–gene and gene–environment interactions for a wide variety of different complex phenotypes, such as incidence of human diseases and other clinical endpoints (Gilbert-Diamond and Moore, 2011). Recently this method has been applied even for studies of pharmacogenomics for thiopurines and methotrexate (Dervieux et al., 2012; Dorababu et al., 2012a,b; Kim et al., 2012), even if its application to this field is still limited and there is great potential for discovery, in particular to detect and elucidate multilocus genotypes associated with genome-wide studies of complex pharmacogenetic phenotypes.

## Conclusion

Consideration of genetic biomarkers can improve therapy of pediatric ALL: the role of *TPMT* genetic polymorphism on mercaptopurine induced toxicity in children with ALL has been clearly defined and clinical guidelines have been developed to tailor treatment with this medication on the basis of TPMT status. Multilocus genotypes have been shown to be able to increase the amount of interindividual variability in a phenotype of clinical relevance explained: for example, the incidence of severe GI toxicity during consolidation therapy has been shown recently to be independently related to *TPMT*, *SLCO1B1*, and *PACSIN2* genetic polymorphisms. Identification, proper testing, and validation of multilocus genotypes hold great potential in further refining the clinical utility of pharmacogenetics to improve treatment of children with ALL by reducing treatment-related adverse events.

## Conflict of Interest Statement

The authors declare that the research was conducted in the absence of any commercial or financial relationships that could be construed as a potential conflict of interest.
